# Healthcare and telehealth use among U.S. immigrants during the COVID-19 pandemic

**DOI:** 10.3389/fpubh.2024.1422343

**Published:** 2024-10-24

**Authors:** Merianne Rose T. Spencer, Sunjung Yoon, Youngeun Lee, Arturo Vargas Bustamante, Jie Chen

**Affiliations:** ^1^Department of Health Policy and Management, School of Public Health, University of Maryland, College Park, MD, United States; ^2^Department of Health Policy and Management, Fielding School of Public Health, University of California, Los Angeles, Los Angeles, CA, United States; ^3^Hospital and Public Health InterdisciPlinarY Research (HAPPY) Lab, School of Public Health, University of Maryland, College Park, MD, United States

**Keywords:** delay of care, telehealth, immigrant, healthcare access, COVID-19

## Abstract

**Introduction:**

Recent studies have documented the negative impact of the COVID-19 pandemic on low-income families, rural and underserved areas, and racial and ethnic minority populations. However, less is known about immigrants’ healthcare access and utilization, including telehealth use. This study investigated disparities in healthcare access and utilization by immigration status among adults aged 18–64 years during the COVID-19 pandemic.

**Methods:**

This cross-sectional study pooled data from the 2020 and 2021 National Health Interview Survey (NHIS). Multivariable logistic regression models were used to estimate the variation of healthcare access and utilization by citizenship and nativity status. Immigration status (U.S.-born citizen, naturalized U.S. citizen, and non-citizen) served as the key independent variable. Healthcare access measures were dichotomized indicators of whether individuals had delayed medical care either due to cost or due to COVID-19. Healthcare utilization measures included whether individuals visited a doctor, used the emergency room, or had a telehealth visit during COVID-19.

**Results:**

Compared to U.S.-born citizens, non-citizen immigrants were more likely to delay medical care due to cost (adjusted OR = 1.375, [95%CI: 1.137, 1.662]), less likely to visit a doctor (adjusted OR = 0.71, [95%CI: 0.617, 0.817]), or visit an emergency room (adjusted OR = 0.756, [95%CI: 0.635, 0.901]). Non-citizen immigrants were less likely to have a telehealth visit (either video or phone visits) during COVID-19 (adjusted OR = 0.634, [95%CI: 0.557, 0.723]).

**Discussion:**

Compared to U.S.-born citizens, lower healthcare and telehealth utilization persisted among non-citizen immigrants during the pandemic.

## Introduction

The coronavirus disease of 2019 (COVID-19) placed an enormous economic and social burden on disadvantaged populations across the globe including those in the United States (1, 2). A wealth of research describes how the COVID-19 has negatively impacted low-income families (3), rural and underserved areas (4), and racial and ethnic minorities (2, 5, 6), yet less is known about the effect of the COVID-19 pandemic on healthcare access and use among U.S. immigrants (5, 7).

Immigrants face unique challenges that can lead to suboptimal use of high-quality healthcare. It is estimated that immigrants represent 14% of the U.S. total population (8). Immigrants are more likely to be uninsured, where almost half (50%) of undocumented immigrants and one in five documented adults (18%) were uninsured in 2023, compared to 6% of naturalized immigrants and 8% of U.S.-born adults (9). Immigrants are at greater risk of having less access to healthcare due to lack of health insurance coverage, experiencing difficulties navigating the U.S. healthcare system, having limited English proficiency, and earning less disposable income (10–12). During COVID-19, immigrants were vulnerable to financial and relationship hardship such as unemployment, housing insecurities, access to resources, and family relationship strains, among other demands (13). Finally, studies have demonstrated that racial and ethnic minorities, as well as individuals residing in rural areas encountered difficulties in using telehealth services and other digital resources (14, 15). Furthermore, research has shown variation by insurance type (e.g., public, private insurance) and telehealth use among those with lower socioeconomic status (16), and in areas with higher rates of poverty (17, 18) where less is known about the potential digital divide among immigrants.

Telehealth and the use of digital health usage have increased due to the COVID-19 pandemic (19). Telehealth use, which includes the combined use of internet and information technology for health purposes, played a crucial role in helping facilitate social distancing while bolstering health access to health services across the country without overcrowding facilities during a surge of COVID-19 infections and hospitalizations (16). While telehealth use has resulted in overall improvements in healthcare access and overall efficacy in health interventions (20), the use of telehealth is more limited in rural areas and among patients with lower incomes (21). For instance, lacking resources and supplies as well as accessibility to medical facilities and internet access can result in disparities in access and use of telehealth services (22, 23). Additionally, benefits may vary by socioeconomic status due to limitations in health literacy, education, and technical competence (24, 25). Thus, understanding the impact of COVID-19 and use of telehealth services is an important aspect of focus for understanding the U.S. immigrant population.

To our knowledge, no study has used nationally representative survey data to explore healthcare access and utilization among U.S. immigrants during the pandemic with a particular focus on telehealth. Using the National Health Interview Survey (NHIS), this study investigated healthcare utilization including telehealth use among adults ages 18–64 by U.S. immigration status during the COVID-19 pandemic. Given the longstanding disparities in healthcare access that immigrants encountered, we hypothesized that immigrant disparities, i.e., lower healthcare access and utilization among non-citizens, might persist or worsen during the pandemic years.

## Methods

### Data source and study population

Respondents of the 2020 and 2021 National Health Interview Survey (NHIS) aged 18–64 were included in this analysis. The NHIS is a nationally representative household survey of the U.S. civilian population that collects information on health status, health-related behaviors, and healthcare access and utilization. Details of the NHIS survey, data, and methodology are publicly available on the National Center for Health Statistics website (26, 27). This survey collects data using computer-assisted personal interviewing. Due to disruptions from the COVID-19 pandemic, data collection procedures changed where, from April 2020 to June 2020, all interviews were conducted by telephone, rather than in-person. The response rate of households for the 2020 sample was 50.7%. The final sample of adults’ response rate was 48.9%. The NHIS provides sample weights to analytically account for the low response rates and oversampling of particular subgroups during participant recruitment. As such, this analysis accounted for the complex sample design of the NHIS by dividing the sampling weights by the total years pooled (i.e., 2 years), as suggested in NHIS documentation available on the Centers for Disease Control and Prevention’s website (28).

### Dependent variables

This study included five outcome measures on healthcare access and utilization. This study dichotomized responses (yes/no) from five survey questions of the NHIS: (1) unmet needs due to COVID-19, (2) unmet needs due to cost, (3) visited a doctor or other healthcare professional in the past 12 months, (4) visited the emergency room in the last 12 months (dichotomized as 0 times or 1 visit or more), and (5) had an appointment by video or by phone in the last 12 months.

### Key independent variable

Immigration status served as the independent variable of interest, which used NHIS responses on birth and citizen status to represent three categories: U.S.-born citizen, naturalized U.S. citizen (i.e., foreign-born U.S. citizens), and non-citizen. Although the NHIS does not distinguish this, we assume that immigration status includes both lawfully present and undocumented immigrants in the United States.

### Other independent variables

In alignment with the Andersen Healthcare Utilization framework (29) and the literature on immigrant healthcare utilization (30, 31), this study included predisposing, enabling, and health need factors: age group (18–24, 25–34, 35–44, 45–54, and 55–64 years), sex (male and female), ratio of income to poverty (0.00–1.24, 1.25–1.99, 2.00–3.99, and 4.00+), marital status (married and not married), education level (less than high school degree, has a high school degree, and has some college or more), race and ethnicity (non-Hispanic White, non-Hispanic Black, non-Hispanic Asian, non-Hispanic other/multiple race, and Hispanic), insurance status (uninsured, private, and public), self-reported health status (poor or fair, good, and excellent), urban/rural status (urban and rural), and household region (Northeast, Midwest, South, and West).

### Analytical approach

Descriptive analyses of weighted frequencies, means, and percentages were generated by immigration status (U.S.-born citizen, foreign-born U.S. citizen, and non-citizen). Wald tests were conducted to evaluate differences in immigration status categories (Table 1). Unadjusted logistic regression models were fitted to evaluate differences between the independent variable (U.S.-born citizenship, foreign-born citizenship, and non-citizen) and the outcomes of interest (Table 2). Multivariable regressions were used to estimate the odds of avoiding medical care due to COVID-19, delaying medical care due to cost, seeing a doctor in the past year, visiting an emergency room in the past year, and having used telehealth services (either video or phone visit) to see a clinician in the past year. The adjusted models controlled for age group, sex, poverty, marital status, education level, race/Hispanic origin, self-reported health status, urban/rural status, and region (Table 3). Insurance status was not included in the final models to avoid simultaneity bias (i.e., endogeneity) between insurance status (a predictor of health access) and outcomes measuring health access. A longitudinal study found that recent immigrants were less likely to have private insurance over time and hence had lower probabilities of having a usual place of care, even though there was no direct relationship between immigrant status and usual source of care (32).

**Table 1:** Characteristics by immigration status: National Health Interview Survey, 2020–2021 (total *n* = 40,746).

Characteristic	Citizenship status						
	U.S.-born citizen *n* = 33,530		Foreign-born *n* = 3,975		Noncitizen *n* = 3,241		*p*-value
	*N* = 153,408,129		*N* = 18,779,520		*N* = 18,248,219		
	Mean	95% CI	Mean	95% CI	Mean	95% CI	
Age group							
18–24 years	0.17	[0.16,0.17]	0.07	[0.06,0.08]	0.087	[0.07,0.10]	<0.001
25–34 years	0.23	[0.23,0.24]	0.15	[0.13,0.16]	0.26	[0.23,0.28]	
35–44 years	0.2	[0.19,0.2]	0.23	[0.22,0.25]	0.31	[0.29,0.33]	
45–54 years	0.19	[0.18,0.19]	0.27	[0.25,0.29]	0.23	[0.21,0.25]	
55–64 years	0.21	[0.21,0.22]	0.29	[0.27,0.31]	0.12	[0.11,0.14]	
Sex							
Male	0.5	[0.49,0.5]	0.47	[0.45,0.49]	0.49	[0.47,0.51]	0.083
Female	0.5	[0.5,0.51]	0.53	[0.51,0.55]	0.51	[0.49,0.53]	
Race/Hispanic origin							
Non-Hispanic White	0.7	[0.69,0.72]	0.22	[0.2,0.24]	0.1	[0.09,0.11]	<0.001
Non-Hispanic Black	0.13	[0.12,0.14]	0.13	[0.11,0.15]	0.075	[0.06,0.09]	
Non-Hispanic Asian	0.02	[0.02.02]	0.3	[0.27,0.32]	0.19	[0.17,0.22]	
Hispanic	0.12	[0.11,0.13]	0.34	[0.31,0.37]	0.62	[0.59,0.65]	
Non-Hispanic Other	0.03	[0.03,0.04]	0.017	[0.01,0.02]	0.0088	[0.01,0.01]	
Marital status							
Not married	0.53	[0.52,0.54]	0.32	[0.30,0.34]	0.38	[0.36,0.41]	<0.001
Married	0.47	[0.46,0.48]	0.68	[0.66,0.7]	0.62	[0.59,0.64]	
Education level							
Less than high school degree	0.09	[0.08,0.09]	0.12	[0.11,0.14]	0.34	[0.31,0.37]	<0.001
High school degree	0.26	[0.25,0.27]	0.2	[0.18,0.22]	0.26	[0.24,0.29]	
Some college or more	0.65	[0.64,0.66]	0.67	[0.65,0.7]	0.4	[0.37,0.42]	
Ratio of income to poverty							
0.00–1.24	0.13	[0.12,0.14]	0.11	[0.1,0.13]	0.31	[0.29,0.34]	<0.001
1.25–1.99	0.12	[0.11,0.13]	0.14	[0.12,0.15]	0.22	[0.2,0.24]	
2.00–3.99	0.29	[0.29,0.3]	0.32	[0.3,0.34]	0.25	[0.23,0.27]	
4.00 or more	0.46	[0.45,0.47]	0.43	[0.4,0.45]	0.22	[0.2,0.25]	
Insurance status							
Uninsured	0.10	[0.09,0.1]	0.088	[0.075,0.1]	0.41	[0.38,0.43]	<0.001
Private	0.68	[0.67,0.69]	0.68	[0.66,0.7]	0.39	[0.36,0.42]	
Public	0.22	[0.21,0.23]	0.23	[0.21,0.25]	0.2	[0.18,0.23]	
Self-reported health status							
Poor or fair	0.11	[0.11,0.12]	0.091	[0.08,0.1]	0.12	[0.1,0.14]	<0.001
Good	0.26	[0.25,0.26]	0.29	[0.27,0.31]	0.31	[0.29,0.33]	
Excellent	0.63	[0.62,0.64]	0.62	[0.6,0.64]	0.57	[0.54,0.6]	
Urban–rural status							
Rural	0.15	[0.14,0.16]	0.031	[0.024,0.04]	0.054	[0.04,0.08]	<0.001
Urban	0.85	[0.84,0.86]	0.97	[0.96,0.98]	0.95	[0.92,0.96]	
Household region							
Northeast	0.16	[0.15,0.18]	0.23	[0.2,0.27]	0.16	[0.14,0.19]	<0.001
Midwest	0.23	[0.22,0.25]	0.11	[0.092,0.14]	0.11	[0.09,0.14]	
South	0.38	[0.36,0.4]	0.34	[0.31,0.38]	0.39	[0.35,0.43]	
West	0.22	[0.21,0.24]	0.31	[0.28,0.34]	0.34	[0.3,0.38]	

CI = confidence interval. Estimates are based on household interview of a sample of the civilian, non-institutionalized U.S. population among adults aged 18–64 from the National Health Interview Survey (NHIS) 2020 and 2021. “n” denotes the non-weighted frequency of study participants of the NHIS, and “N” denotes weighted values estimating the prevalence of respondents in a given subcategory based on the NHIS.

**Table 2:** Health care access or utilization by immigration status: United States, 2020–2021 (*n* = 40,746).

Healthcare access or utilization indicator	U.S.-born citizen		Foreign-born U.S. citizen		Non-citizen		*P*-value
	Mean	95%CI	Mean	95%CI	Mean	95%CI	
Delayed medical care due to unmet needs during COVID-19	0.23	[0.23–0.24]	0.23	[0.21–0.25]	0.19	[0.17–0.21]	<0.001
Delayed medical care due to cost	0.10	[0.1–0.1]	0.07	[0.06–0.08]	0.14	[0.12–0.16]	<0.001
Visited a doctor or other healthcare professional in the past 12 months	0.80	[0.79–0.81]	0.80	[0.78–0.82]	0.68	[0.66–0.71]	<0.001
Visited an emergency room visits in the past 12 months	0.18	[0.17–0.19]	0.13	[0.11–0.14]	0.16	[0.14–0.18]	<0.001
Had an appointment by video or by phone in the past 12 months	0.35	[0.34–0.36]	0.34	[0.32–0.36]	0.22	[0.2–0.24]	<0.001

CI, confidence interval. Participant responses to healthcare access and utilization survey questions (which are categorized accordingly in the NHIS documentation), were retrieved from NHIS survey questions and dichotomized into the following five outcomes: (1) delaying medical care due to COVID-19 in the past 12 months; (2) delaying medical care in the past 12 months due to cost; (3) visited a doctor or other healthcare professional in the past 12 months; (4) visited an emergency room (dichotomized as 0 times or 1 visit or more); and (5) had an appointment by video or phone in the past 12 months. Estimates are based on household interview of a sample of the civilian, non-institutionalized U.S. population among adults aged 18–64 from the National Health Interview Survey 2020 and 2021.

**Table 3:** Full logistic regression model output denoted as odds ratios for the indicators on healthcare access and utilization: United States, 2020–2021 (*n* = 40,746).

Indicator	Delayed medical care due to COVID-19		Delayed medical care due to cost		Visited a doctor or other healthcare professional in past 12 months		Visited an emergency room in the past 12 months		Had an appointment by video or by phone because of COVID-19	
	OR	CI	OR	CI	OR	CI	OR	CI	OR	CI
Immigration status										
U.S.-born citizen	Reference		Reference		Reference		Reference		Reference	
Foreign-born U.S. citizen	0.93	[0.82,1.06]	0.93	[0.76,1.15]	1.007	[0.88,1.15]	0.749	[0.64,0.87]	0.94	[0.84,1.06]
Non-citizen	0.90	[0.78,1.04]	1.38	[1.14,1.66]	0.71	[0.62,0.82]	0.756	[0.63,0.90]	0.63	[0.56,0.72]
Age group										
18–24 years	Reference		Reference		Reference		Reference		Reference	
25–34 years	1.35	[1.15,1.584]	1.67	[1.38,2.01]	0.65	[0.57,0.74]	0.93	[0.80,1.07]	1.18	[1.04,1.332]
35–44 years	1.52	[1.30,1.8]	1.26	[1.03,1.54]	0.67	[0.58,0.78]	0.79	[0.68,0.92]	1.30	[1.15,1.48]
45–54 years	1.72	[1.46,2.02]	1.29	[1.05,1.60]	0.92	[0.79,1.07]	0.74	[0.63,0.87]	1.34	[1.18,1.53]
55–64 years	1.88	[1.60,2.20]	1.17	[0.96,1.43]	1.21	[1.04,1.41]	0.71	[0.61,0.82]	1.38	[1.22,1.57]
Sex										
Male	Reference		Reference		Reference		Reference		Reference	
Female	1.69	[1.59,1.81]	1.28	[1.163,1.414]	2.01	[1.872,2.166]	1.29	[1.20,1.39]	1.76	[1.660,1.865]
Race/Hispanic Origin										
Non-Hispanic White	Reference		Reference		Reference		Reference		Reference	
Non-Hispanic Black	0.86	[0.76,0.97]	0.70	[0.60,0.83]	1.46	[1.27,1.69]	1.55	[1.38,1.75]	0.80	[0.72,0.89]
Non-Hispanic Asian	0.79	[0.67,0.92]	0.42	[0.32,0.55]	0.72	[0.61,0.84]	0.72	[0.58,0.90]	0.72	[0.62,0.84]
Hispanic	0.93	[0.83,1.04]	0.86	[0.73,1.01]	0.94	[0.84,1.04]	1.11	[0.98,1.26]	0.84	[0.76,0.93]
Non-Hispanic Other	1.34	[1.12,1.61]	1.09	[0.83,1.43]	1.03	[0.84,1.27]	1.63	[1.35,1.96]	0.99	[0.82,1.17]
Marital status										
Not married	Reference		Reference		Reference		Reference		Reference	
Married	1.00	[0.93,1.07]	0.80	[0.72,0.88]	1.34	[1.23,1.45]	1.01	[0.93,1.10]	1.01	[0.95,1.08]
Education level										
Less than high school degree	Reference		Reference		Reference		Reference		Reference	
High school degree	1.02	[0.89,1.18]	0.90	[0.76,1.07]	1.11	[0.98,1.26]	0.99	[0.86,1.14]	1.26	[1.10,1.44]
Some college or more	1.69	[1.48,1.94]	1.01	[0.86,1.18]	1.37	[1.22,1.55]	0.83	[0.72,0.96]	1.99	[1.74,2.26]
Ratio of income to poverty										
0.00–1.24	Reference		Reference		Reference		Reference		Reference	
1.25–1.99	0.81	[0.70,0.92]	0.10	[0.85,1.17]	0.95	[0.83,1.10]	0.88	[0.78,1.01]	0.96	[0.85,1.09]
2.00–3.99	0.87	[0.77,0.97]	0.84	[0.73,0.96]	1.08	[0.94,1.23]	0.71	[0.63,0.79]	1.04	[0.94,1.15]
4.00 or more	1.10	[0.98,1.24]	0.44	[0.38,0.50]	1.42	[1.25,1.61]	0.59	[0.52,0.66]	1.21	[1.08,1.36]
Self-reported health status										
Poor or fair	Reference		Reference		Reference		Reference		Reference	
Good	0.57	[0.51,0.64]	0.60	[0.52,0.68]	0.52	[0.45,0.61]	0.49	[0.44,0.55]	0.48	[0.44,0.53]
Excellent	0.39	[0.35,0.43]	0.40	[0.31,0.41]	0.39	[0.34,0.46]	0.31	[0.28,0.34]	0.30	[0.27,0.33]
Urban–rural status										
Rural	Reference		Reference		Reference		Reference		Reference	
Urban	1.21	[1.09,1.34]	0.99	[0.85,1.14]	0.98	[0.87,1.11]	0.89	[0.80,0.99]	1.60	[1.43,1.79]
Household region										
Northeast	Reference		Reference		Reference		Reference		Reference	
Midwest	0.76	[0.68,0.86]	1.45	[1.20,1.75]	0.97	[0.85,1.11]	1.07	[0.95,1.20]	0.72	[0.64,0.82]
South	0.74	[0.66,0.83]	1.72	[1.44,2.05]	0.93	[0.82,1.06]	1.03	[0.92,1.15]	0.74	[0.67,0.83]
West	0.93	[0.82,1.05]	1.48	[1.21,1.80]	0.86	[0.76,0.98]	0.99	[0.88,1.12]	1.07	[0.95,1.20]

CI, confidence interval; OR, odds ratio. Immigration status was based on survey responses to country of birth and citizenship status. Estimates are based on household interview of a sample of the civilian, non-institutionalized U.S. population among adults aged 18–64 from the National Health Interview Survey 2020 and 2021. Results have adjusted for adult age (18–24, 25–34, 35–44, 45–54, 55–64), sex, income ratio to poverty (0–1.24; 1.25–1.99; 2.00–3.99; 4.00 or more), marital status (not married; married), education level (less than high school degree; high school degree; some college or more), race/Hispanic origin; self-reported health status (poor or fair; good; excellent), urban–rural status; household region.

For the sensitivity analysis, we fitted multivariable logistic regression models that only accounted for age group, sex, and race/ethnicity, given these are non-modifiable covariates in the analysis. Results were similar and available upon request. Survey weights were applied in STATA SE 17.0 to calculate appropriate estimates of population parameters.

## Results

This study included 40,746 adult respondents of the 2020 and 2021 NHIS for a weighted frequency of 153,408,129 U.S.-born citizens (33,530 unweighted), 18,779,520 foreign-born U.S. citizens (3,975 unweighted), and 18,248,219 non-citizens (3,241 unweighted).

Summary statistics of the population by immigration status are presented in Table 1. A greater percentage of non-citizens were Hispanic compared to U.S.-born citizens (62% versus 12%). A larger percentage of non-citizens had less than a high school degree (34%) compared to U.S.-born citizens and foreign-born U.S. citizens (8.8 and 12%, respectively). Non-citizen immigrants were more likely to be uninsured compared to U.S.-born citizens and foreign-born citizens (41% versus 10 and 9%, respectively).

U.S.-born and foreign-born citizens had a significantly higher percentage of having delayed medical care due to COVID-19 than non-citizens (23% compared to 19%). Non-citizens were also more likely to delay medical care due to cost (14% compared to 10 and 7% for U.S.-born citizens and foreign-born citizens, respectively), less likely to visit a doctor (68% vs. 80%) or had a virtual visit (34–35% compared to 22%) during the pandemic year.

Adjusted odds ratios (aOR) of logistic regression models were provided in Table 3. Non-citizens had decreased odds of reporting having delayed medical care due to COVID-19 compared to U.S.-born citizens, but the difference was not significant (aOR = 0.903 [95% CI: 0.782, 1.042]). Additionally, non-citizens were significantly less likely to have a telehealth visit with a physician, nurse, or other clinician during COVID-19 than U.S.-born citizens (aOR = 0.634 [95% CI: 0.56, 0.72]). Compared to U.S.-born citizens, non-citizens had greater odds of delaying medical care due to cost in the past year (aOR = 1.375 [95% CI: 1.14, 1.66]). Compared to U.S.-born citizens, non-citizens were less likely to see a doctor in the past 12-months (aOR = 0.71 [95% CI: 0.62, 0.82]). Both foreign-born U.S. citizens and non-citizens were less likely to use an emergency department in the past 12 months compared to U.S.-born citizens.

## Discussion

Our study suggests that compared to U.S.-born citizens, non-citizen immigrants were associated with a greater likelihood of delaying medical care due to cost and a lower likelihood of visiting a doctor or emergency room in the previous year during the COVID-19 pandemic. These findings are consistent with the healthcare patterns of immigrants pre-COVID-19 pandemic (33). These results suggest that lower healthcare access and use among U.S. immigrants persisted during the pandemic despite flexibilities approved by Congress to offer free COVID-19 testing, treatment, and vaccination (34).

The reduced healthcare access and use among non-citizen immigrants can be attributed to various systematic barriers, such as being ineligible for public benefit programs like Medicaid (35). In addition, unstable insurance coverage, limited support in navigating the healthcare system (particularly or those with limited English proficiency), and fears of disclosing immigration status have been identified in previous research as major barriers to access care among immigrants (36, 37).

Lower healthcare access and utilization among immigrants could also be partly explained by the “healthy immigrant effect” (38–40) and social determinants of health (41). Immigrants were more likely to work in “essential” jobs and reside in areas with high COVID-19 cases; this can result in immigrants being among the first populations exposed to COVID-19 (42).

The findings on telehealth suggest that non-citizen immigrants are associated with a decreased likelihood of having a virtual appointment to speak to a doctor or other clinician in the past year. These findings might be highlighting differences in access and use of digital resources (including the digital divide), availability of telehealth services in languages other than English, and barriers to healthcare access that are a result of low income or low socioeconomic status (43, 44). Immigrant households have the highest rates of lacking internet access. Without reliable high-speed internet access, people may have difficulty scheduling virtual appointments that have both video and audio (rather than just audio), accessing medical information online, or communicating with healthcare providers (45).

Health literacy may also play a significant role in effectively utilizing digital resources for healthcare (46, 47). However, it is worth noting that evidence of virtual care use by limited English proficiency has been inconclusive. Studies have shown that patients, especially those with limited English proficiency, often prefer in-person care because of the anxiety experienced from conducting self-evaluations without direct medical guidance (48). On the other hand, studies have reported that immigrants viewed telehealth more favorably compared to in-person care because they could bypass the logistical challenges of in-person visits, which can be particularly difficult for people with limited English proficiency (49).

Trust is another important factor that affects telehealth use among immigrant populations in the United States (49–51). Individuals may have concerns about the confidentiality and security of their medical information when accessing telehealth services, particularly if they are not familiar with the technology or do not speak English fluently (14). Studies have analyzed the hurdles encountered by immigrants with limited English proficiency to connect with providers who speak Spanish and other languages during the pandemic (52). Thus, a lack of trust can hinder the uptake of telemedicine use among immigrant populations (53).

Our study has several limitations. First, the data collection process of the NHIS experienced significant shifts during the COVID-19 pandemic. In 2020, most household interviews transitioned to phone-based interviews. This shift affected respondent participation rates and might have introduced measurement bias. However, as part of our sensitivity analyses, we compared the results when focusing solely on the 2021 NHIS survey, and we found that the findings were similar. Hence, we included both the 2020 and 2021 NHIS surveys to maximize the power available in the study to account for predisposing, enabling and health need factors. Second, the cross-sectional design of the NHIS limits drawing inferences about causal relationships of the study findings. The relationships discussed in this paper are statistically significant associations and should not be interpreted as causal relationships. The cross-sectional design of the NHIS also limits our ability to explore mediators, such as the effect of insurance status on immigration status and their access and use to health services; for example, studies have reported a temporal relationship in the length of time since immigrating to the country and the ability of attaining and maintaining health insurance over the year (32, 54, 55), yet having insurance directly affects healthcare access and utilization. Future studies exploring these relationships during COVID-19 could further our understanding of healthcare care patterns in utilization among immigrants. Third, we were not able to separate video and phone from telehealth use. Studies suggested that racial and ethnic minority patients were more likely to use phones rather than virtual visits. These disparities in virtual telehealth might be more pronounced by immigration status (14, 56). Additional qualitative studies examining the differences between video telehealth visits and phone telehealth visits are necessary in order to consider the potential differences between these two forms of telehealth use. Fourth, we were unable to clarify legal immigrant status due to data limitations. Additional studies focusing on differences between documented and undocumented non-citizens are necessary to understand the breadth of access barriers that may be impacting these immigration groups and communities. Finally, the study focused on NHIS participants who were aged 18–64; while these findings were weighted to generate nationally representative estimates, it is important to note that these findings are not generalizable to children and those 65 years and older, as well as those who would not participate in the NHIS survey (e.g., those who are institutionalized, military overseas).

## Conclusion

This study offers a national perspective on immigrants aged 18–64 and their patterns of healthcare access and utilization during the COVID-19 pandemic. This study touches on the importance of telehealth access among immigrants, particularly non-citizen immigrants. These findings can offer additional insights about the barriers to access quality healthcare, as well as reasons to invest and develop innovative telehealth resources that can bridge the digital divide and provide equitable and affordable healthcare access to all. Future research should identify differences by documentation status among non-citizen immigrants.

## Data Availability

Publicly available datasets were analyzed in this study. This data can be found: https://www.cdc.gov/nchs/nhis/2020nhis.htm, https://www.cdc.gov/nchs/nhis/2021nhis.htm.
